# Improving Rheological and Mechanical Properties of Various Virgin and Recycled Polypropylenes by Blending with Long-Chain Branched Polypropylene

**DOI:** 10.3390/polym13071137

**Published:** 2021-04-02

**Authors:** Sascha Stanic, Thomas Koch, Klaus Schmid, Simone Knaus, Vasiliki-Maria Archodoulaki

**Affiliations:** 1 Institute of Materials Science and Technology, TU Wien, Getreidemarkt 9, 1060 Vienna, Austria; thomas.koch@tuwien.ac.at (T.K.); vasiliki-maria.archodoulaki@tuwien.ac.at (V.-M.A.); 2 Pergan GmbH, Schlavenhorst 71, 46395 Bocholt, Germany; dr.schmid@pergan.com; 3 Institute of Applied Chemistry, TU Wien, Getreidemarkt 9, 1060 Vienna, Austria; simone.knaus@tuwien.ac.at

**Keywords:** reactive extrusion, long-chain branching, recycled polypropylene, blends, upcycling, rheology, mechanical properties

## Abstract

Blends of two long-chain branched polypropylenes (LCB-PP) and five linear polypropylenes (L-PP) were prepared in a single screw extruder at 240 °C. The two LCB-PPs were self-created via reactive extrusion at 180 °C by using dimyristyl peroxydicarbonate (PODIC C126) and dilauroyl peroxide (LP) as peroxides. For blending two virgin and three recycled PPs like coffee caps, yoghurt cups and buckets with different melt flow rate (MFR) values were used. The influence of using blends was assessed by investigating the rheological (dynamic and extensional rheology) and mechanical properties (tensile test and impact tensile test). The dynamic rheology indicated that the molecular weight as well as the molecular weight distribution could be increased or broadened. Also the melt strength behavior could be improved by using the two peroxide modified LCB-PP blends on the basis of PODIC C126 or PEROXAN LP (dilauroyl peroxide). In addition, the mechanical properties were consistently enhanced or at least kept constant compared to the original material. In particular, the impact tensile strength but also the elongation at break could be increased considerably. This study showed that the blending of LCB-PP can increase the investigated properties and represents a promising option, especially when using recycled PP, which demonstrates a real “up-cycling” process.

## 1. Introduction

The worldwide production of plastics has experienced great growth in the last decades and therefore the consumption of plastics has also increased more and more. As such, plastics and their products are already being promoted as the material of the 21st century. The three largest areas in which plastic products play an important role are the packaging industry with 40%, building and construction with 20%, and the automotive industry with 10%. For these and other application segments, the plastics that are used can be divided into different polymers. These include polypropylene (PP), polyethylene (PE), polyvinyl carbonate (PVC), polystyrene (PS), polyethylene terephthalate (PET) and others [1].

Isotactic polypropylene (PP) is one of the most popular and most universally applicable plastics worldwide with one of the largest share in global plastic production next to polyethylene (PE) [1]. PP is used in a wide range of applications from plastic pipes to various packaging applications and injection molding. Due to its excellent properties, such as good mechanical properties, high melting temperature, high chemical resistance or low density, PP is used in the aforementioned applications [2]. In general, the recycling of plastics and thus also of PP is becoming increasingly important due to the enormous production and consumption of plastics. In the literature there are different approaches for the solution of plastic waste such as mechanical recycling, energy recovery and landfill [3,4]. In particular, mechanical recycling is environmentally friendly and represents an economically viable practice, where theoretically a saving of 20–50% of the market price is achieved compared to the virgin material [5,6,7,8]. Various methods have been tried out in the literature to promote the quality of the recycled plastic. The basic idea is to add other material during the melting process such as virgin polymers, fillers, fibers, compatibilizers etc. [9,10,11,12,13,14,15,16,17,18,19,20,21].

However, the use of linear PP (L-PP) is limited because it has a relatively low melt strength and shows no strain hardening, which is particularly important in processes such as thermoforming, foaming and blow molding [22,23,24]. According to the literature, the introduction of long chain branching improves the properties of L-PP like a more pronounced shear-thinning effect or strain hardening, which leads to the effect that the melt strength increases significantly [25,26,27,28,29,30]. There are different methods to produce long-chain branched PP (LCB-PP) such as direct synthesis using metallocene catalysis [31,32], electron beam irradiation [33,34,35] or reactive extrusion [36,37,38,39,40]. Reactive extrusion is the most popular method because it requires no solvents, allows short reaction times and involves relatively low infrastructure costs [41,42]. In addition to the introduction of long-chain branching, various concurrent reactions take place in the case of polypropylene during reactive extrusion. Mainly, chain scission (β-scission) is expected in the case of PP, which leads to the formation of unsaturated short chain segments as a result [43,44]. Due to the use of peroxides, this phenomenon tends to become more common and is known as controlled rheology PP (CR-PP). This leads to a reduction in viscosity and to a reduction in molecular weight as well as a narrowing of the molecular weight distribution [45,46]. The use of peroxides can lead to further side reactions during reactive extrusion such as recombination disproportionation of peroxide radicals. The temperature used also plays an important role in reactive extrusion in the case of PP. Higher temperatures lead to β-scission and disproportionation being favored to some extent, thus negatively affecting the introduction of LCB. For this reason, reactive extrusion is mostly carried out at a temperature of 180 °C, whereas higher temperatures are used in industry and the balance shifts in the direction of chain scission.

Nonetheless, not only can the production of LCB-PP lead to an increase in melt strength. In order to achieve a higher melt strength also the blending with L-PP and LCB-PP is possible to obtain a significant increase of strain hardening. According to Stange et al. [47], a small amount of LCB-PP influences the rheological properties significantly, especially the elongation behavior. The same behavior was concluded by Fang et al. [48], which reported that adding LCB-PP into L-PP improved the melt strength resulting in a strain-hardening behavior. In addition to the rheological properties of L-PP/LCB-PP blends, according to McCallum et al. [49] not only could the melt strength be increased but also the mechanical properties as well. These studies have been carried out mainly with virgin PP materials.

The aim of this work is to investigate the rheological and mechanical properties of blends consisting of PP respective recycled PP and 10 wt.% LCB-PP blended at an extrusion temperature of 240 °C to simulate industry-related processes. The novel LCB-PPs (based on dimyristyl peroxydicarbonate (PODIC C126) and dilauroyl peroxide (PEROXAN LP)), which were produced by reactive extrusion in a previous study in the research group and investigated for their mechanical and rheological properties, were used as blend material in this work [50]. In order to investigate the blend properties of these two LCB-PP materials as potential masterbatch material, a virgin linear PP but also a commercial available high melt strength polypropylene (HMS-PP) with a content of 10 wt.% were additionally used in this work for better comparison. For the experiments in this study, two different virgin PPs were used, which covered a melt flow rate (MFR) range between 8–21 g/10 min. Besides the virgin material, three different recyclates consisting of flakes from yoghurt cups, coffee caps and buckets were blended with the respective LCB-PPs. To the best of our knowledge, no papers have been published in which recycled PP and their blends with LCB-PP were analyzed.

## 2. Materials and Methods

### 2.1. Materials

Two different isotactic PP homopolymers were used for the study, which were supplied by Borealis (Vienna, Austria). The type of HD 601CF (PP3; MFR value of 8 g/10 min at 230 °C and 2.16 kg) is normally used as film resin for chill roll processes and the second virgin PP, HF 700SA (PP4; MFR value of 21 g/10 min) can be used for different injection molding applications. Additionally, three different PP were collected from household post-consumer waste, which consisting of flakes from coffee caps (MFR = 14.3 g/10 min), which was supplied by Walter Kunststoffe GmbH (Wels, Austria), flakes of yoghurt cups (thermoformed PP; MFR = 5.3 g/10 min) and flakes of buckets (injection-molded PP; MFR = 31.9 g/10 min). The yoghurt cups and buckets were self-collected.

The LCB-PPs, which were used for the blends, were based on HC 600TF (PP2) appropriated for thermoforming applications, which was also supplied by Borealis (Vienna, Austria), and the peroxides PODIC C126 (dimyristyl peroxydicarbonate; 10 h half-life time at 48 °C) or PEROXAN LP (dilauroyl peroxide; 10 h half-life time at 61 °C) from Pergan GmbH (Bocholt, Germany). The choice of peroxide content was investigated on the basis of a preliminary study, which examined different concentrations from 10 to 200 mmol/kg PP of the respective peroxides on virgin material. An increase in melt stiffness as well as an increase in mechanical properties, tensile strength and impact strength, could be observed [50]. These two modified materials have an MFR value of 7.7 g/10 min for PODIC C126 and an MFR value 6.0 g/10 min for PEROXAN LP at 230 °C and 2.16 kg weight. In general, the PP2 with an MFR value of 2.8 g/10 min was used as blend material in this study to get a better comparison between the blends with L-PP and LCB-PP. In addition, the Daploy™ WB140HMS from the company Borealis (Vienna, Austria) with an MFR value of 2.1 g/10 min served as a comparison material of a high melt strength polypropylene (HMS-PP).

### 2.2. Sample Preparation

The two LCB-PPs were produced using a three-zone single-screw extruder, Extron 18–26−1.5, with a screw diameter of 18 mm and a ratio L/D = 25:1 (Extron Engineering, Ltd., Akaa, Finland). The rotational speed was 70 rpm and the temperature profile for the three independent temperature zones (feeding zone/extrusion zone/die zone) was 165/180/220 °C. The pure material (PP2) and the respective peroxide (PODIC C126 or PEROXAN LP) with a quantity of 10 wt.% were mixed directly and added to the extruder.

**Figure 1:**
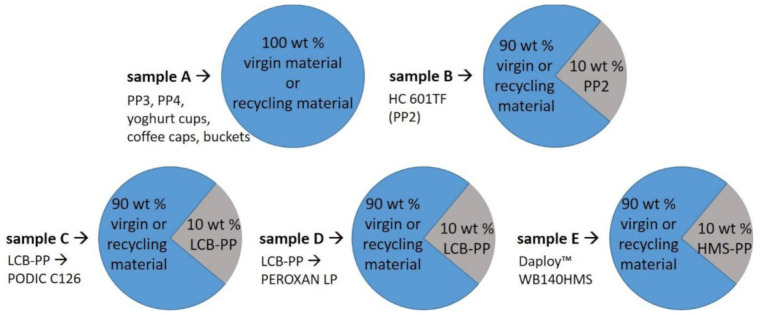
Schematic blend composition and specification of all series.

For this study and the production of the blend, the respective pure materials and the investigated mixing ratios are shown schematically in Figure 1.

For better mixing during extrusion (blend production), the three post-consumer waste materials and both LCB-PPs were shredded before use with a universal cutting mill, Pulverisette 19 (Fritsch, Idar-Oberstein, Germany), equipped with a 4 mm sieve insert. The extrusion or blending of these samples were performed at a temperature profile of 165/240/240 °C and also a screw rotation of 70 rpm. The polymer strands produced were then ground again with the Pulverisette 19 for further production steps.

### 2.3. Melt Flow Rate (MFR)

With a manual testing device from Karg Industritechnik (Krailling, Germany), MeltFlow basic, the MFR measurements were performed in accordance with DIN EN ISO 1133 method A under a load of 2.16 kg and a temperature of 230 °C [51].

### 2.4. Differential Scanning Caloremtry (DSC)

The thermal behaviour of all materials (virgin/recycling materials and their blends) after extrusion at 240 °C was investigated by using TA Q2000 differential scanning calorimeter (DSC) from TA-Instruments (Newcastle, DE, USA). We inserted 5–10 mg of each sample in aluminium pans and the measurements were carried out under a nitrogen atmosphere. The samples were heated from room temperature to 200 °C at a rate of 10 K/min, cooled to room temperature and heated a second time also at a rate of 10 K/min to 200 °C. The analysis software TA Universal Analysis was used to determine the crystallisation temperature (*T*_c_) and from the second heating the melting temperature (*T*_m_) as well as the enthalpy of fusion (Δ*H*_m_). From the enthalpy of fusion the degree of cristallinity *X*c was calculated using 207 J/g as the enthalpy of a fully crystalline PP.

### 2.5. Fourier Transform Infared (FTIR) Measurements

The Fourier transform infrared (FTIR) spectroscopy measurements were carried out at room temperature with a spectrometer (Tensor 27) of the company Brucker (Billerica, MA, USA), which is equipped with an ATR diamond (DuraSample IR II) with single reflection. The measurement covered with a number of 16 scans and a wave number range of 600–4000 cm^−1^ with a 4 cm^−1^ resolution.

### 2.6. Rheology Properties

The characterisation of the polymer melt and its rheological properties was carried out by using dynamic rheology and extensional rheology measurements. The specimens for the dynamic rheology measurements were made by compression molding (Collin P 200 P, Maitenbeth, Germany) at a temperature of 240 °C and a pressure of 30 bar. The dimensions for the round samples were 25 mm in diameter and 1.2 mm thick. For the extensional rheology measurement stripes with dimensions of 20 × 8 mm were necessary, which in form of squares were produced by compression molding with dimensions of 60 × 60 × 0.8 mm.

The dynamic rheology respectively the frequency sweep test was done on a plate-plate system with a diameter of 25 mm via Anton Paar (Graz, Austria) MCR 302 rheometer, which was equipped with a CTD 450 heating chamber, under nitrogen at 230 °C. The frequency range was selected from 628 to 0.01 rad/s, the deformation rate increased logarithmically over the selected range from 1% to 2%, and the gap size of the plate-plate system was 1 mm. Also the extensional rheology measurements were done at the Anton Paar MCR 302 rheometer, which was equipped with a Sentmanat Extensional Rheometer (SER-HPV 1, Xpansion instruments, Tallmadge, OH, USA). Three different strain rates ($\dot{\varepsilon }$ = 5; 1; 0.1 s^−1^) were used at a temperature of 180 °C and additionally the corresponding start up curves were measured with a plate-plate system with a diameter of 25 mm at 0.001 and 0.1 s^−1^ shear rates.

### 2.7. Mechanical Properties

Tensile and tensile impact strength tests were used for the determination of the mechanical properties. The required test specimens were produced using Haake Mini Lab II (Thermo Fisher, Waltham, MA, USA), a twin screw extruder, in combination with Haake Mini Jet II injection molding equipment from Thermo Fisher Scientific (Waltham, MA, USA). The extrusion was carried out at a temperature of 240 °C and a screw rotation of 100 rpm. For the injection molding process, the mold temperature was set at 90 °C, the pressure was 350 bar with an injection time of 10 s.

The test machine for the tensile test (Zwick 050, ZwickRoell GmbH and Co. KG, Ulm, Germany) was used with a test speed of 10 mm/min and was equipped with a 1 kN load cell and an extensometer. The test specimens for these measurements corresponded to the standard ISO 527-2-5A [52].

For the tensile impact strength test the specimens were notched on both sides with a Notch-Vis from Ceast and tested according to ISO 8256/1A [53] on an Instron Ceast 9050 (2 J hammer; cross head mass = 15 g, Instron, Darmstadt, Germany).

### 2.8. Scanning Electron Microscopy (SEM)

For the morphological investigation of the respective samples, the fracture surfaces of the impact tensile test samples were analyzed using scanning electron microscopy (FEI Philips XL30, Hillsboro, OR, USA). The individual samples were coated with gold prior to imaging (Agar Sputter Coater B7340, Stansted, UK).

## 3. Results

### 3.1. Melt Flow Rate

In general, the MFR value gives a first good approximation of melt behavior and the process ability of polymer blends. It is generally known that the MFR value, especially for PP, can increase very strongly as a result of β-scission and the associated increase in chain mobility, but can also be reduced by long chain branching or cross-linking [54,55,56].

**Figure 2:**
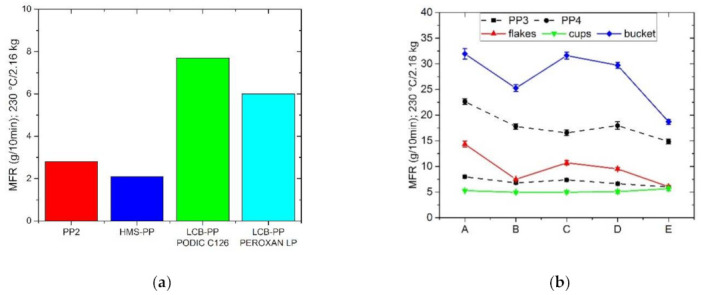
Melt flow rate (MFR) values of the materials, which was used for blending (**a**) as well as all starting materials and their blend series (**b**); measured at 230 °C with 2.16 kg.

Figure 2a shows the MFR values of the blending materials, which amounted for PP2 = 2.8 g/10 min, LCB-PP modified by PODIC C126 = 7.7 g/10 min, LCB-PP modified by PEROXAN LP = 6.0 g/10 min. Figure 2b represents the MFR values of all materials after the extrusion temperature of 240 °C. It can be clearly seen that the use of blends reduces the MFR values. In particular, for the starting materials with a high MFR value such as PP4 (virgin granulate) but also for the flakes or bucket a reduction is visible. For the two materials with a lower MFR value (PP3 and cups) a slight reduction or at least keeping the values constant can be observed. However, as these values do not increase due to the addition of LCB-PP, which show a higher MFR than cups, it can be assumed that the mixing rule is not the reason for the reduction of the MFR values.

### 3.2. Dynamic Rheology Properties

**Figure 3:**
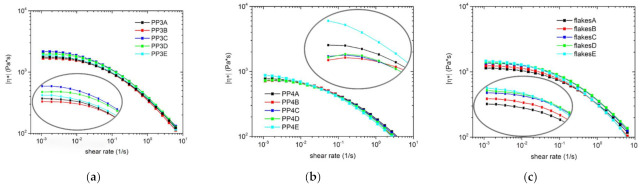

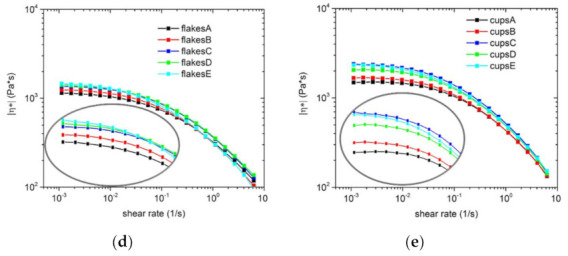
Complex viscosity of all samples (polypropylenes PP3 (**a**) and PP4 (**b**), flakes (**c**), cups (**d**), bucket (**e**)) and their blends; measured at 230 °C.

Rheology measurements have been shown in literature to be a good tool of identifying molecular structures and to be very sensitive to changes in them [36,57,58,59,60,61,62,63,64]. Several studies have investigated the rheological behavior of blends with linear PP and branched PP or PE and have shown that these blends have a higher shear thinning effect or strain hardening compared to linear PP [47,48,49,65]. The Figure 3 show the viscosity curves, which were measured at 230 °C to achieve better comparability with the MFR values, of the respective virgin materials and the blends. The viscosity curves of the respective materials are very close to each other, so these curves were additionally plotted larger in the low shear range in order to get a better overview. For the series with lower MFR (PP3, flakes and cups) it was found that the use of LCB-PPs or HMS-PP resulted in a pronounced increase in viscosity. While the blends with L-PP only achieved a slight increase in viscosity for the recycled materials, the curve progression of virgin granules could only be kept constant. No clear trends could be observed for the two series with higher MFR (PP4 and buckets). On the one hand, no changes between the individual viscosity curves are clearly visible in the PP4 series blends and on the other hand, an opposite trend could be observed in the recycled bucket and its blends compared to the other recyclates. In this case, the blend with L-PP shows an increase in the viscosity curve, whereas the blends with LCB-PPs and HMS-PP show a small reduction.

**Figure 4:**
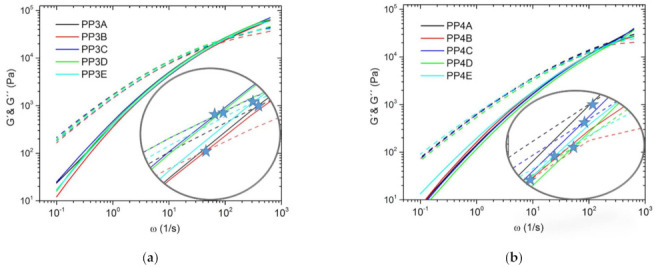
Storage modulus (*G*′) lines and loss modulus (*G*″) dashed lines of the series PP3 (a) and PP4 (b) and their blends; the stars represent the crossover point of the two curves (*G*′ and *G*″) of the respective blend series; measured at 230 °C.

**Figure 5:**
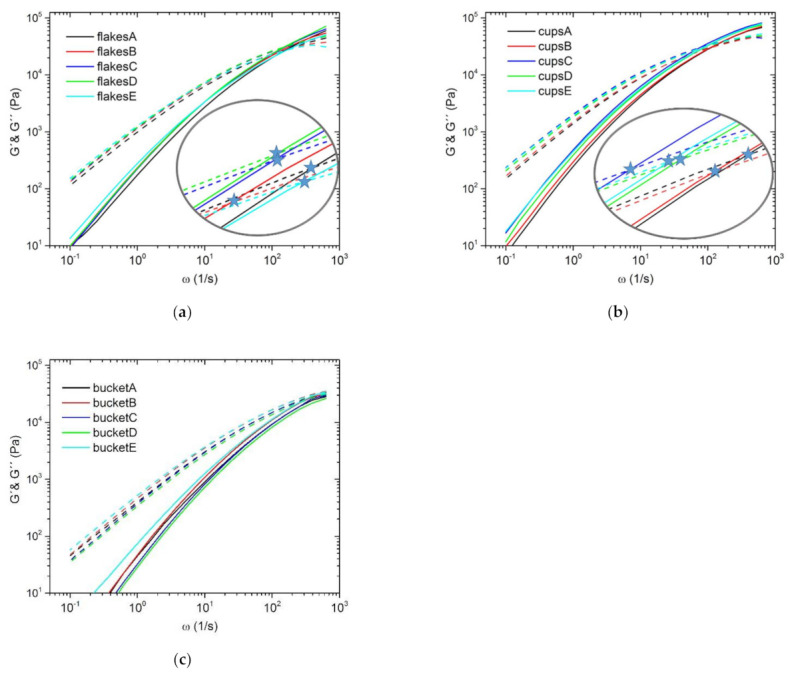
Storage modulus (*G*′) lines and loss modulus (*G*″) dashed lines of the flakes (**a**), cups (**b**), bucket (**c**) and their blends; the stars represent the crossover point of the two curves (*G*′ and *G*″) of the respective blend series; measured at 230 °C.

In addition to the complex viscosity, the storage modulus (*G*′) and the loss modulus (*G*″) are important parameters in dynamic rheology, which provide information about viscous or elastic effects depending on the shear rate [66,67]. In Figure 4 and Figure 5 the respective curves of the associated series are shown. For a better representation of the crossover points, this area of the curves was displayed larger and additionally the intersection points were marked with a star for each graph. Figure 4 shows the curves of the two virgin materials PP3 (a) and PP4 (b) and their blends. If both pictures are compared, it becomes clear that the curves and the cross points of *G*′ and *G*″ are shifted from PP4 to higher frequencies, which is attributable to the high MFR values. As the curves are so close to each other in nearly all cases and the crossing point of *G*′ and *G*″ partly overlap, the respective values are given in Table 1.

Figure 5 additionally shows the series of recyclates (flakes (a), cups (b) and bucket (c)). Comparing the different recyclates with each other, the difference between the crossing points of *G*′ and *G*″ is even more pronounced. It can be seen that with increasing MFR value the crossing point shifts to higher frequencies. This is most pronounced with the bucket. Here no intersection point could be determined under the given measurement conditions because it was outside the measurement range. However, the values of the crossing points of the other two series (flakes and cups) are also listed additionally in Table 1.

The position or change of the crossover point can be used to determine the change in molecular weight (*M*_W_) or molecular weight distribution (MMD) [68,69,70,71,72,73]. If the crossover point shifts to lower frequencies, this represents the presence of branched molecules with longer relaxation time. In contrast, the vertical shift of the crossover point to lower *G* values results from a broadening of the MMD. The values of the crossover frequency *ω*_C_ and crossover modulus *G*_C_ are given in Table 1. By comparing the individual series and their blends with each other, it can be seen that both the *ω*_C_ values and the *G*_C_ values are lower than the virgin/recycled material used. All in all, this could mean that the use of blends, which have been applied in this work, can increase the molecular weight and broaden the molecular weight distribution compared to the respective starting materials.

### 3.3. Extensional Rheology

It is known that extensional rheology plays an important role in the detection of long-chain branching and the associated changes in molecular structure [74,75,76,77]. It has been shown in various studies that the melt strength, which is also referred to as strain hardening, can be increased compared to linear PP [78,79,80,81,82]. The increased melt strength of materials is very important and desired for many manufacturing processes such as thermoforming, film casting, film blowing, foaming, and so on [83,84].

**Figure 6:**
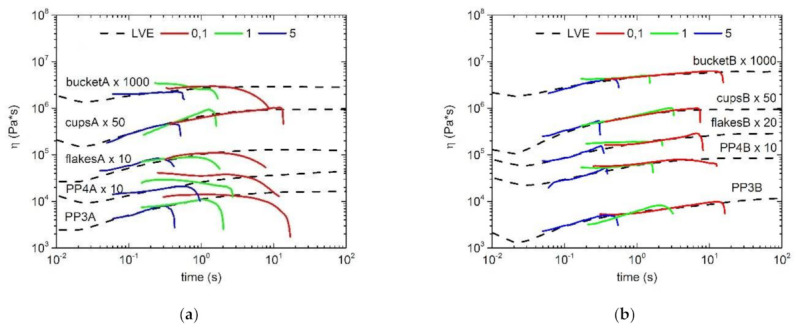
Extensional rheology of all curves for the virgin materials (**a**) and with the PP2 blends series (**b**).

**Figure 7:**
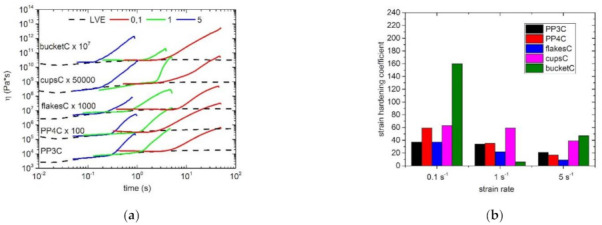

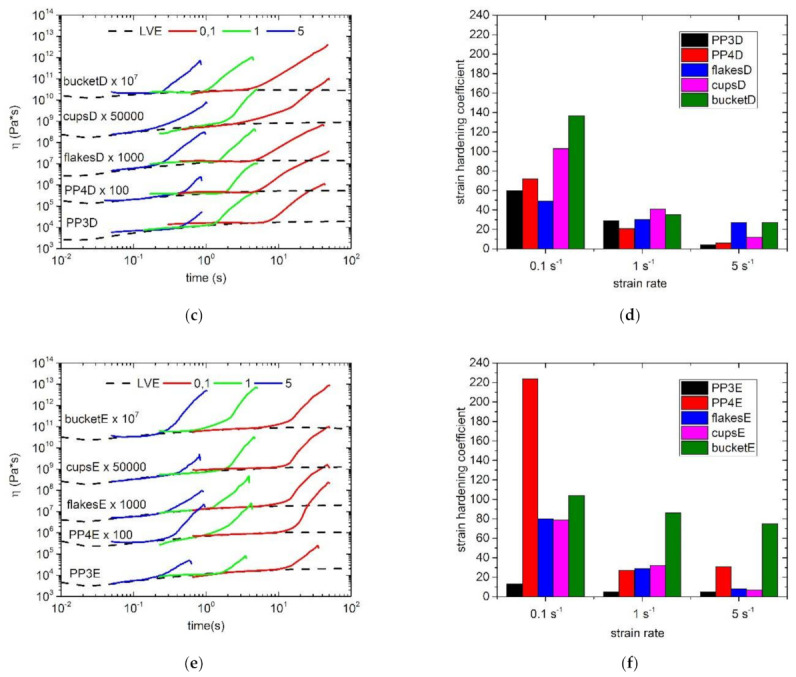
Extensional rheology curves of all samples with the blend series of long-chain branched polypropylene (LCB-PP) dimyristyl peroxydicarbonate (PODIC C 126) (**a**), with LCB-PP dilauroyl peroxide (PEROXAN LP) (**c**) and HMS-PP (**e**); Strain Hardening Coefficient of all samples with the blend series of LCB-PP PODIC C 126 (**b**), with LCB-PP PEROXAN LP (**d**) and HMS-PP (**f**).

The following figures show the starting materials and the respective blends using PP2, the two LCB-PPs and HMS-PP. The dashed lines correspond to the linear viscoelastic start-up curve (LVE), which was measured by shear rheology and multiplied by the Trouton factor to obtain the extensional viscosity [85]. The presence or absence of melt strength (strain hardening) of all materials were measured with three different Hencky strain rates ($\dot{\varepsilon }$ = 5; 1; 0.1 s^−1^). As expected, the virgin materials showed no deviation of the respective strain curves from the LVE and thus no strain hardening could be determined in all cases (Figure 6a). The same result was reflected in the blends with a content of 10 wt.% PP2, which is represented in Figure 6b. Again, no deviation and thus no strain hardening could be achieved. In contrast, the two blends with the respective LCB-PPs and HMS-PP showed a significant deviation from the LVE curve for all three different strain rates. In these cases, a significant strain hardening and associated increased melt strength behavior can be seen for the LCB-PP with PODIC C126 in Figure 7a, for LCB-PP with PEROXAN LP in Figure 7c and for HMS-PP in Figure 7e.

In order to make a better statement or distinction about the strain hardening behavior and thus the melt strength of the blends with the two LCB-PPs and HMS-PP, the strain hardening coefficient (SH) of the respective blends was determined. This represents the maximum strain viscosity η(t) at the strain hardening rate in relation to the strain viscosity of the LVE curve η_0_(t). The results are represented in Figure 7b,d,f. It can generally be seen that with increasing strain rate, the strain hardening coefficient continuously decreases for both blended sets. Comparing the two blend sets with each other, no general statement can be made about the results with PODIC C126 or PEROXAN LP. However, this is clear evidence that the use of LCB-PPs produced by ourselves in laboratory scale as blends can successfully increase melt strength (Figure 7b). Especially with recycled materials, a significant increase in melt strength was observed when using PEROXAN LP LCB-PP (Figure 7d). Here the strain hardening coefficient tends to be higher than the values of the virgin material (PP3 and PP4). In order to be able to make a better statement about the two LCB-PPs, the melt strength of the blends with HMS-PP and their strain hardening coefficients were also investigated (Figure 7f). On the one hand, the same trend was observed again, that the melt strength of the respective series decreased with increasing strain rate and, on the other hand, that the blends with LCB-PPs, with the exception of PP4 and a strain rate of 0.1 s^−1^, can keep up with the blends with HMS-PP in terms of melt stiffness.

All in all, the melt strength properties of the relevant samples, especially those of the recycled materials, can be increased and thus new areas of application can be found.

### 3.4. Thermal Characterization

All materials, including the complete mixtures of the different blends, exhibit single melting and crystallization peaks. The temperature of melting (*T*m) and crystallization (*T*c) as well as the crystallinity of all blends and their virgin material are listed in the Table 2. To determine the crystallinity, the melting curves were integrated from 80 °C to the temperature of return to baseline after melting (The diagrams of the second heating are presented in Figure S1 in the Supplementary Material). Except for the buckets which consist of a PP copolymer and has a much broader melting peak with lower peak temperature. In that case the integration starts at 50 °C. Regarding the crystallization temperature there is no clear trend visible, in most cases there is no strong shift by the addition of PP2 and the LCB-PPs. The same behavior was reported from Tabatabaei et al. [86] for blends containing 10 wt.% LCB-PP and more. Adding of HMS-PP results in every series in the highest crystallization temperature, which indicates a nucleating effect. This is also visible by a slight increase in crystallinity for the mixtures of PP3, PP4, flakes and cups with HMS-PP. The blends from the buckets show a different behavior, there occurred a notable increase of the crystallization temperature if the second components are added. This is not unlikely if primarily homopolymer type PP is added to a PP copolymer. Interestingly, the blends of the bucket with the LCB-PPs show a 10% increase of crystallinity. It should be respected that the melting curves of PP3C, PP4A-D and flakesC and E contain a small shoulder at a temperature between 149–152 °C, which it the typical melting temperature of the β—phase of PP. In principle this should be considered if someone calculates the crystallinity from melting curves because a superposition of melting of the β—phase and recrystallization to the α—phase takes place. But this shoulder is really small and the calculated crystallinities do not show outliers, so it can be neglected.

### 3.5. Mechanical Properties

**Figure 8:**
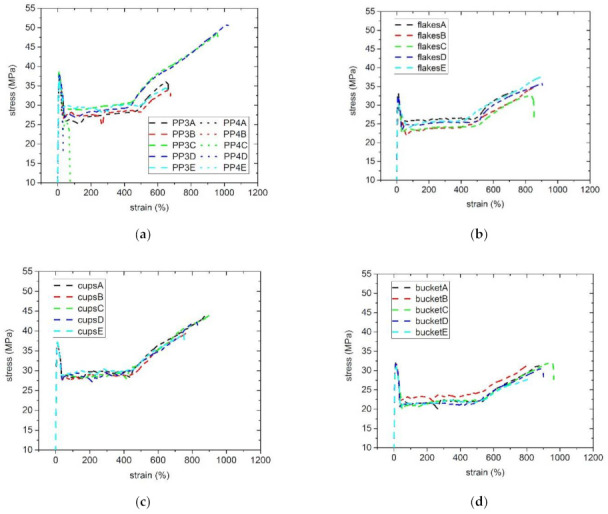
Stress-strain curves of all samples (PP3 and PP4 (**a**), flakes (**b**), cups (**c**), bucket (**d**)) obtained from tensile test.

The determination of mechanical properties of each material consists of tensile and tensile impact strength test, whereby both tests are good methods to analyze the material properties in dynamic and quasi-static loading. In particular, the tensile test and its data like modulus of elasticity, elongation at break and tensile strength gives a good insight into the general deformation behavior of polymers and, therefore, allows general statements to be made about the influence of material modification [87,88,89]. Figure 8 illustrates the stress–strain curves for all materials, which were obtained from tensile test.

Additionally, Table 2 displays the tensile properties, including the tensile modulus (*E*_t_), the elongation at break (*ε*_B_) and the tensile strength (*σ*_m_). It could be observed, that the tensile strength, which is defined as first maximum of the stress–strain curve according ISO 527-1, was kept constant for all blends compared to their virgin materials [52]. Also, the observation of the tensile modulus did not show any significant change and the values are constant within a certain range. However, the elongation at break showed a change as a result of the use of blends. It was found that the sets of blends consisting LCB-PP had an increase and at least a constant level in comparison to the virgin materials. For example, the values of PP3 increased by 33% and 46%, respectively, through the blending with LCB-PP, but also a significant increase of 23% was achieved with the recycled flakes.

**Figure 9:**
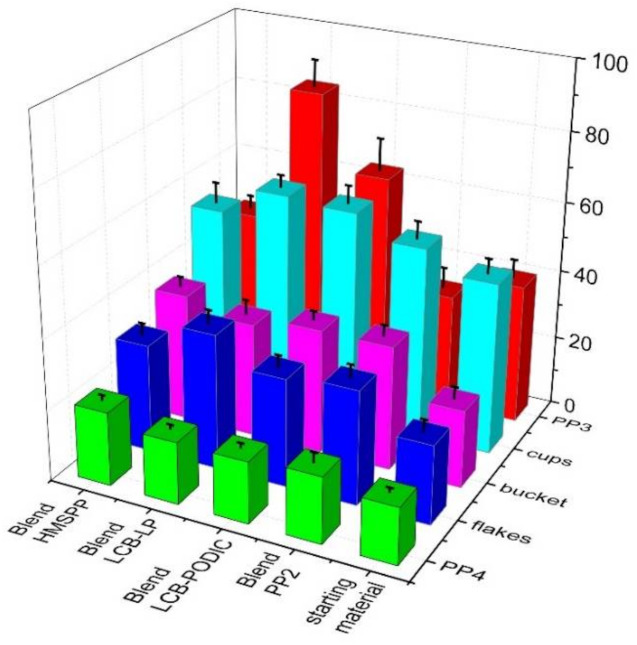
Tensile impact test of all samples.

The results of the tensile impact strength test are shown in Figure 9. Here it can be clearly seen that the use of blends leads to an increase in impact tensile strength. An increase can be seen in the blending with a pure material (PP2), with the exception of PP3, as well as in the use of LCB-PP. In addition, it is evident that the addition of LCB-PP can further increase the results. This is especially the case for the materials of PP3, flakes and cups, whereby the LCB-PPs modified with PEROXAN LP are even more pronounced than the respective blends. The respective series with HMS-PP blends also showed an improvement in impact strength compared to the starting materials. However, if we compare the blends of the two LCB-PPs and the HMS-PP with each other, we see that the values are almost constant for the series with PP4 and bucket. However, for the materials with the lower MFR values (PP3, flakes and cups), the LCB-PPs show an increase compared to the HMS-PP. In particular, the blends with LCB-PP, which were modified with PEROXAN LP, are outstanding.

### 3.6. Scanning Electron Microscopy (SEM)

**Figure 10:**
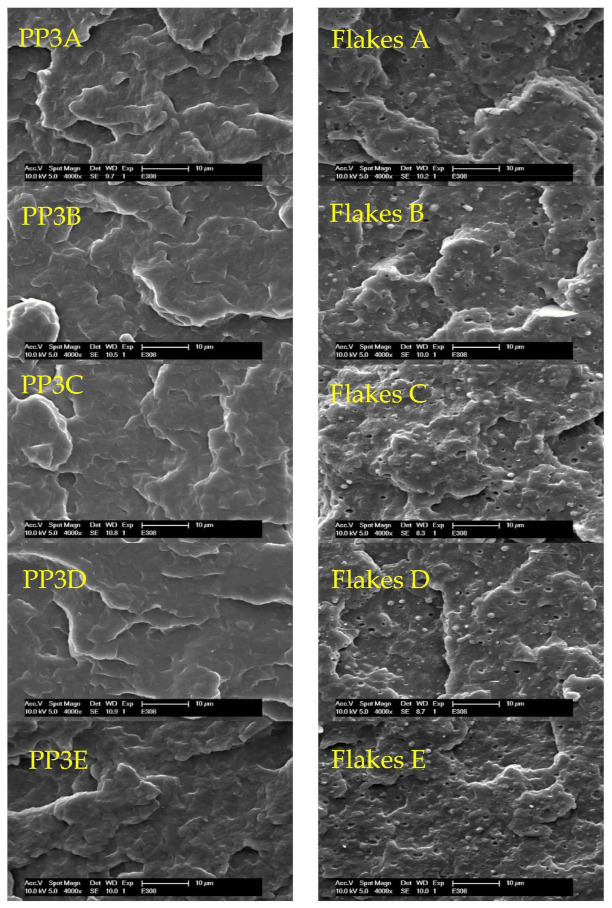
Scanning electron microscope (SEM) images of the virgin material of PP3 and the recycled flakes (**A**) and the blend series on basis of PP2 (**B**), LCB-PP with PODIC C126 (**C**), LCB-PP with PEROXAN LP (**D**), HMS-PP (**E**).

In addition to the previous results, the mixing behavior of the respective starting materials and the corresponding blends was analyzed using scanning electron microscopy (SEM, Figure 10). For this purpose, the fracture surfaces of the specimens resulting from the impact tensile test were examined. The following pictures show examples of the virgin material PP3 and the blends with PP2, PP-LCB (PODIC C126 and PEROXAN LP) as well as HMS-PP and, for comparison, the recycled flakes of the coffee caps and their corresponding blends.

If a view is taken of the virgin material PP3A and its blends (PP3B-PP3E), no representative difference can be seen between the individual images of the respective morphology. Therefore, it can be assumed that the materials used for the blends in this quantity (10 wt.%) do not influence the morphological structure and thus the miscibility of the blend is guaranteed. This reflects the results of the DSC measurements which have already been described.

In addition, PP3 and the flakes of the recycled coffee caps have almost the same MFR value, which makes it possible to compare the two materials very well.

A morphological difference between the two starting materials PP3 and flakes of coffee caps can be seen clearly. It can be observed that the recycled post-consumer material (flakesA) already has some polymeric particles in the fracture surface. However, no changes could be detected through the blending process with L-PP (flakesB), LCB-PP on basis of PODIC C126 (flakesC), LCB-PP on basis of PEROXAN LP (flakesD) or HMS-PP (flakesE). This means that no additional particle formation in the morphological structure was recognizable and therefore the blends used did not play a role in the miscibility of the recycled material. This in turn means that there is no phase segregation at the 10 wt.% blend content used and it is, therefore, very easy to process.

**Figure A1:**
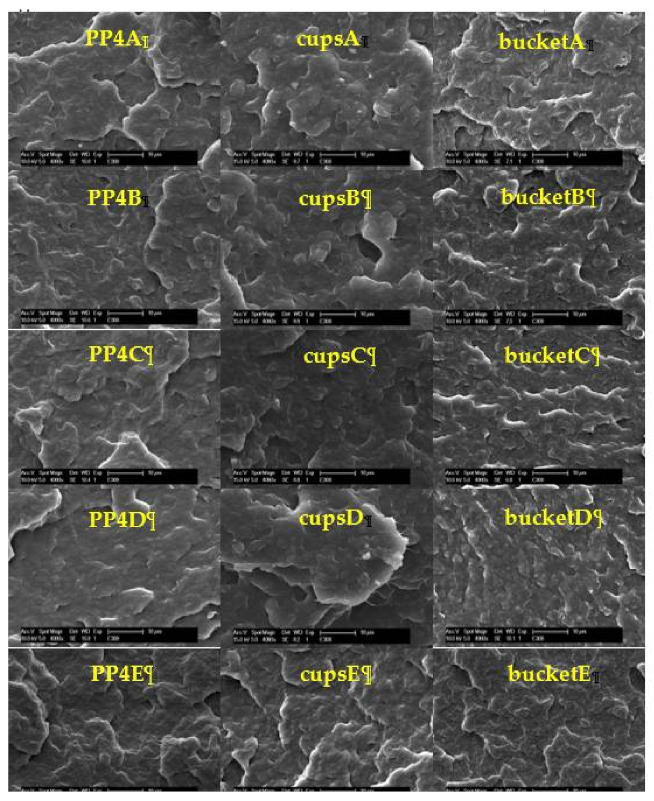
SEM images of the virgin material of PP4 and the recycled flakes of cups and bucket (**A**) and the blend series on basis of PP2 (**B**), LCB-PP with PODIC C126 (**C**), LCB-PP with PEROXAN LP (**D**), HMS-PP (**E**).

These results, which were just described for the two materials at PP3 as well as for the recycled flakes of the coffee caps, were also evident for the other materials. Although there was a large difference in the MFR values between PP4 or the flakes of the recycled buckets and the respective blended materials, no phase segregation was observed here either. These studies of the starting materials with pure PP4, recycled flakes of yoghurt cups and recycled flakes of the bucket and their different blends are shown in Figure A1.

### 3.7. Characterization of Modified Polymers Long-Chain Branched Polypropylenes (LCB-PP)

**Figure 11:**
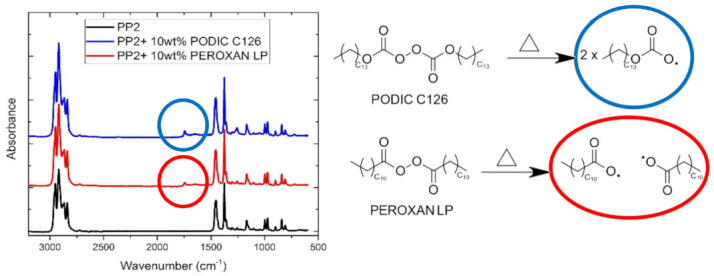
Fourier transform infrared (FTIR) spectra of the unmodified PP and the two LCB-PPs.

The branching mechanism of PODIC C126 and PEROXAN LP was examined by using FTIR. It is well known in the literature that the modification or branching with different components such as HDDA (1,6-hexanediol diacrylate) or MAH (maleic anhydride) by using FTIR can be demonstrated clearly [37,59,90,91]. Figure 11 shows the FTIR spectra of the unmodified PP and the two LCB-PPs each modified with 10 wt.% peroxide. The two modified PPs show an absorption band at 1743 cm^−1^ compared to the unmodified PP. This band describes the stretching vibration of a carbonyl group of an ester, which is generated by the introduction of long-chain branching and the use of peroxides [25,92,93].

**Figure 12:**
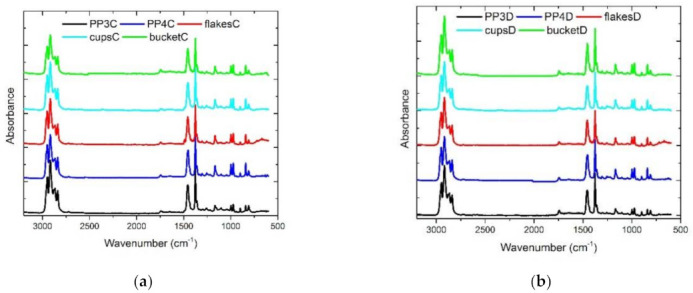
FTIR spectra of the blend series with LCB-PP (**a**) on basis of PODIC C126 and (**b**) on the basis of PEROXAN LP.

To characterize whether the long-chain branches of the LCB-PPs based on PODIC C126 and PEROXAN LP are still present after blending at an extrusion temperature of 240 °C, the FTIR spectra of the respective materials (virgin/recycled materials) were additionally examined. The spectra of the blends with 10 wt.% LCB-PP are shown in the Figure 12. As shown in Figure 11, the characteristic band at 1743 cm^−1^ for the decomposition products of the peroxides could be detected by introducing the long chain branches. This band can be shown for LCB-PP based on PODIC C126 in Figure 12a and for LCB-PP based on PEROXAN LP in Figure 12b after blending with the respective virgin/recycled materials. This finally supports the statement that the LCB-PP blends can be processed very well at an extrusion temperature of 240 °C with virgin/recycled material, since on the one hand the long chain branching can be detected in the FTIR and on the other hand the LCB-PP blends, show no impurities at the molecular level as already indicated by the SEM results.

## 4. Conclusions

In this study, the influence of blends on virgin PP (HD 601CF and HF 700SA) and recycled PP (flakes of yoghurt cups, coffee caps and buckets) was investigated. For this purpose, two LCB-PPs were used, which were produced and investigated in a previous work in the research group. The two LCB-PPs were prepared by reactive extrusion and by using 10 wt.% peroxides (PODIC C126 and PEROXAN LP) and served as masterbatches in this work. To investigate the influence of these a virgin blend material, a linear PP (HC 600TF) and a commercially available HMS-PP (Daploy™ WB140HMS) were also used as blend materials. The blends each contained 10 wt.% of the second component.

The starting materials that were investigated and blended had different initial MFR values ranging from 5 g/10 min to an MFR of 32 g/10 min, thus covering a wide range of different PPs. By using the respective second blend components, a reduction or at least a constant MFR value could be achieved. In general, the starting materials with higher MFR values, such as the recycled buckets or recycled flakes, but also the vrigin PP (HF 700SA), showed a greater reduction of the MFR value than the starting materials with lower MFR values.

The rheology measurements were carried out at 230 °C to obtain a better comparison of the complex viscosity versus the MFR values. The series, using virgin PPs, showed different curves of complex viscosity. In the case of HD 601CF, the addition of the two LCB PPs and the commercial HMS PP led to an increase in viscosity in the low-frequency range, whereas the linear PP as blending material led to a decrease. In the series, using HF 700 SA, the curves are so close together that no significant change in viscosity can be assumed. Also in the series with the different recycled materials, no uniform trend could be observed. For the series of the flakes of yoghurt cups and coffee caps the same trend was seen by the use of the different blend components. It led in both cases to an increase in viscosity compared to the starting materials. In the case of the flakes of the bucket, again, a different trend is shown and no increase in viscosity of the blends with the two LCB-PPs and the commercially available HMS-PP could be achieved.

In addition to the viscosity curves, the curves of the storage and loss moduli were also studied, as well as their crossover points. In principle, an increase in molecular weight as well as a broadening of the molecular weight distribution could be achieved by using the blend materials compared to the respective starting material. However, the flakes of the recycled bucket were an exception. In the specified measuring range an approximation between the storage modulus curve and the loss modulus curve can be recognized but no corssover point could be measured for this series and thus no statement could be made.

However, in order to determine whether strain hardening also occurs due to the use of the respective blending materials, the elongational rheology was also investigated in addition to the dynamic rheology. Here, a large difference was found between the linear PP as blend material compared to the two LCB-PPs and the commercially available HMS-PP. The starting materials as well as the series blended with the linear PP showed no strain hardening and thus no melt stiffness. However, the blend series with the two LCB-PPs and the commercial HMS-PP showed strain hardening throughout. Comparing the blends with each other and contrast the series with the linear PP with those of the LCB-PPs, the two blend series with the respective LCB-PPs have the advantage that a melt stiffness occurs with them and thus a significant strain-hardening effect occurs. If one compares these two series with the blend series of the commercial HMS-PP, these two can simply keep up in terms of elongation rheology.

In the case of the mechanical properties, especially elongation at break and tensile impact strength, different results could be achieved by using the respective blends, which were consistently better for all blends compared to the starting material. By using commercial PP and HMS-PP, the properties could be increased compared to the virgin materials. However, by blending with LCB-PP this could be increased even more in comparison to the blends with PP or HMS-PP. Finally, for the LCB-PP on the basis of PODIC C126 with the LCB-PP on the basis of PEROXAN LP, both are comparable.

For the thermal characterization, DSC measurements of the starting materials as well as the corresponding blends were performed. The use of the different blends did not result in any changes in the melting temperature compared to the starting material. When analyzing the crystallization temperature, only the blends with HMS-PP showed an increase, whereas the crystallization temperature of the other blends remained uneffected. The degree of crystallization was also not significantly affected by the blending of the individual materials. The only exceptions were the two blends with LCB-PP based on PODIC C126 or PEROXAN LP, which were added to the flakes of buckets. Here an increase in crystallinity could be detected.

The SEM measurements clearly showed the miscibility of the individual blends with the respective start materials. In particular, when comparing the SEM photos of the virgin/recycled material with the blend series, no influence of the blends could be detected and thus no particle contamination could be verified. In addition, by using FTIR measurements, it could be verified that the modified LCB-PPs used for blending showed the characteristic band for the carbonyl group of an ester at 1743 cm^−1^ for all virgin/recycled materials even after extrusion at 240 °C.

According to theses results, by using 10 wt.% LCB-PP based on PODIC C126 or PEROXAN LP, the properties of recyclates can be upgraded, leading to an actual upcycling of these materials. This makes it possible to specifically the use the recyclates in other areas of application where a higher melt strength is desired or required.

## Figures and Tables

**Table 1:** Results of crossover point (G′ = G″) from dynamic rheological measurements at 230 °C.

Sample	*ω*_C_ (rad/s)	*G*_C_ (kPa)	*M*w/MMD
PP3A	102	24.52	
PP3B	78	20.26	*M*w ↑, MMD ↑
PP3C	80	23.51	*M*w ↑, MMD ↑
PP3D	81	22.96	*M*w ↑, MMD ↑
PP3E	100	24.09	*M*w ↑, MMD ↑
PP4A	255	21.18	
PP4B	170	15.29	*M*w ↑, MMD ↑
PP4C	238	19.37	*M*w ↑, MMD ↑
PP4D	232	18.87	*M*w ↑, MMD ↑
PP4E	201	16.96	*M*w ↑, MMD ↑
FlakesA	177	32.47	
FlakesB	124	25.02	*M*w ↑, MMD ↑
FlakesC	150	30.16	*M*w ↑, MMD ↑
FlakesD	152	31.01	*M*w ↑, MMD ↑
FlakesE	170	27.47	*M*w ↑, MMD ↑
cupsA	123	31.86	
cupsB	106	29.61	*M*w ↑, MMD ↑
cupsC	76	30.00	*M*w ↑, MMD ↑
cupsD	93	31.22	*M*w ↑, MMD ↑
cupsE	90	31.14	*M*w ↑, MMD ↑

**Table 2:** Crystallization temperature (*T*_c_), melting temperature and crystallinity (2nd heating) of all samples obtained from differential scanning calorimetry (DSC) measurements; mechanical properties (tensile modulus, elongation at break and tensile strength) obtained from tensile test.

Sample	*T*_c_ (°C)	*T*_m_ (°C)	*X*_C_ (%)	*E*_t_ (MPa)	*ε*_B_ (%)	*σ*_m_ (MPa)
PP3A	115	167	50	2039 ± 139	689 ± 65	38.7 ± 0.5
PP3B	119	169	49	1848 ± 115	650 ± 105	36.5 ± 0.7
PP3C	121	167	50	2181 ± 140	921 ± 77	38.9 ± 0.3
PP3D	115	165	50	1904 ± 108	1008 ± 34	38.1 ± 0.5
PP3E	129	168	53	2037 ± 81	681 ± 57	36.9 ± 0.3
PP4A	118	164	50	1960 ± 18	23 ± 4	37.1 ± 0.5
PP4B	118	164	48	1898 ± 151	35 ± 4	37.0 ± 0.5
PP4C	120	165	50	1980 ± 164	77 ± 25	36.9 ± 0.3
PP4D	117	165	50	1950 ± 125	26 ± 6	36.7 ± 0.7
PP4E	128	167	50	1966 ± 53	43 ± 5	36.8 ± 0.4
FlakesA	121	167	42	1864 ± 55	748 ± 85	32.8 ± 0.6
FlakesB	121	165	43	1436 ± 35	863 ± 48	29.8 ± 0.4
FlakesC	122	165	44	1717 ± 51	917 ± 38	31.7 ± 0.2
FlakesD	121	165	44	1733 ± 66	916 ± 49	32.2 ± 0.3
FlakesE	125	166	45	1502 ± 123	912 ± 21	30.5 ± 0.4
cupsA	129	167	51	1794 ± 143	859 ± 39	37.1 ± 0.3
cupsB	129	167	50	1913 ± 188	758 ± 35	37.5 ± 0.4
cupsC	129	167	50	1708 ± 123	913 ± 32	36.9 ± 0.4
cupsD	129	167	51	1818 ± 114	826 ± 29	37.1 ± 0.2
cupsE	130	167	52	1976 ± 188	757 ± 48	37.9 ± 0.3
bucketA	114	151	42	1570 ± 94	892 ± 73	30.9 ± 1.3
bucketB	114	155	44	1537 ± 75	813 ± 129	32.8 ± 0.5
bucketC	114	154	54	1488 ± 81	880 ± 134	30.9 ± 1.1
bucketD	114	153	54	1560 ± 70	864 ± 67	30.4 ± 1.3
bucketE	124	157	45	1476 ± 50	817 ± 69	30.6 ± 1.4

## Data Availability

Not applicable.

## Supplementary Materials

The following are available online at https://www.mdpi.com/article/10.3390/polym13071137/s1, Figure S1: DSC curses of the second heating of the investigated materials; (a) PP3, (b) PP4, (c) flakes of coffee caps, (d) flakes of yoghurt cups, (e) flakes of buckets.
