# Supplementary material for: Improving Rheological and Mechanical Properties of Various Virgin and Recycled Polypropylenes by Blending with Long-Chain Branched Polypropylene
Source: Polymers (Basel). 2021 Apr 2;13(7):1137. doi: 10.3390/polym13071137 (PMC8038188; doi:10.3390/polym13071137)
Supplement: Supplementary file 1 [file polymers-13-01137-s001.pdf]

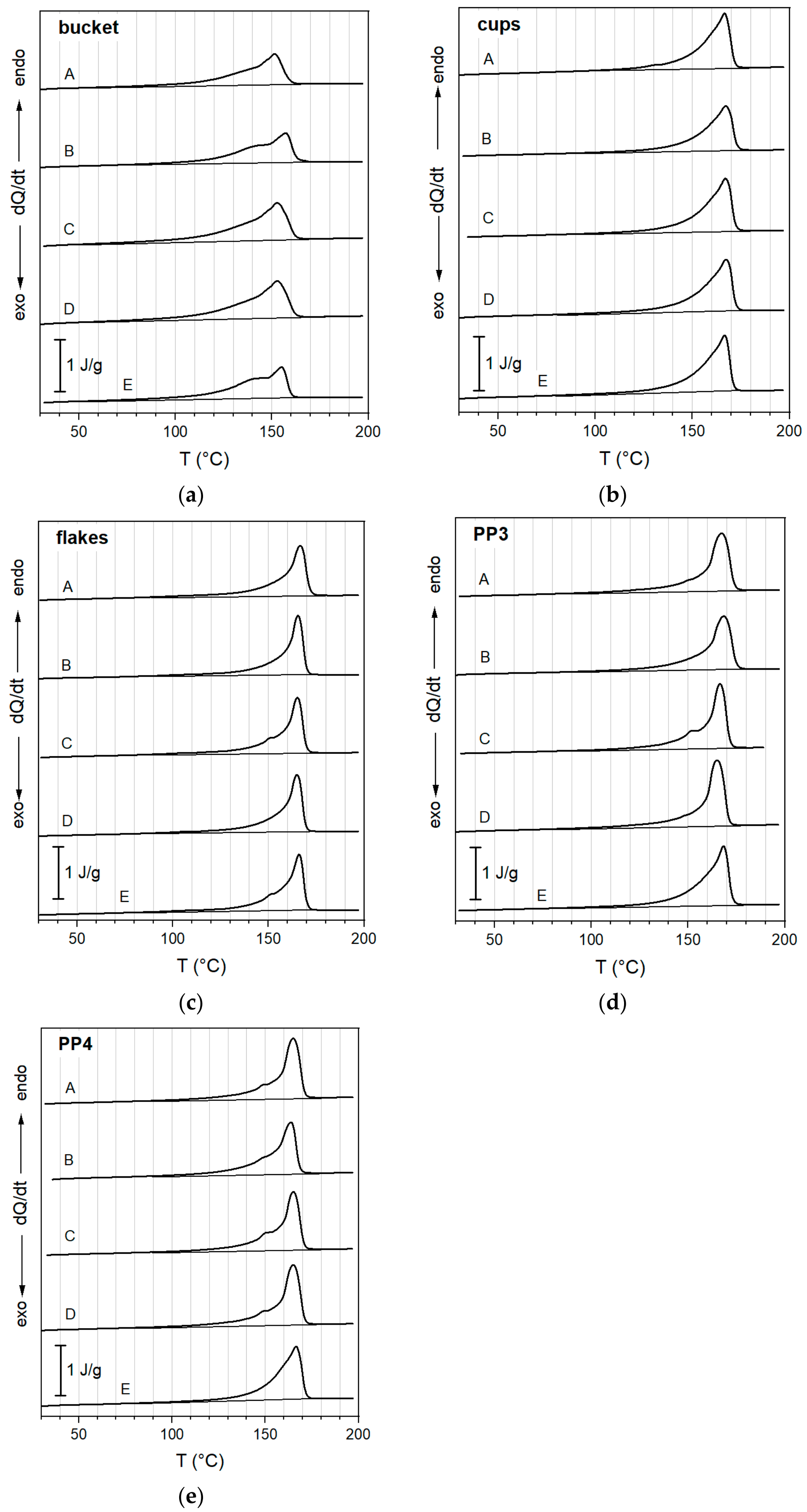

Figure S1: DSC curses of the second heating of the investigated materials; (**a**) PP3, (**b**) PP4, (**c**) flakes of coffee caps, (**d**) flakes of yoghurt cups, (**e**) flakes of buckets
